# Anxiety and Future-Self Clarity: Can Future Thinking Influence Self-Esteem?

**DOI:** 10.1177/00332941251315091

**Published:** 2025-01-16

**Authors:** Jessica Duffy, Grace Salt, Scott N. Cole, Divine Charura, Jennifer Shevchenko

**Affiliations:** School of Education, Language and Psychology, 41872 York St John University, York, UK

**Keywords:** Anxiety, self-esteem, future-self clarity

## Abstract

Possible selves reflect one’s hopes and fears for their future identity. Previous research shows that high anxiety is associated with clearer feared possible selves. However, the mechanism through which clarity could maintain anxiety is relatively unexplored as are questions concerning whether clarity of possible selves could offer new methods of reducing anxiety. The present article aimed to investigate if the relationship between clarity and anxiety is mediated by self-esteem. In addition, the present study aimed to explore the impact of the Best Possible Self-Technique on anxiety. In line with predictions, it was found that the relationship between the clarity of feared possible selves and anxiety was mediated by self-esteem. In addition, a preliminary exploration (using a repeated measures design) showed potential benefits for using a Best Possible Self-Technique in reducing anxiety. Overall, these findings provide insights into a potential mechanism through which the clarity of feared possible selves could influence anxiety, namely, through reducing self-esteem. Also, the findings open new avenues for future interventions designed to reduce anxiety through targeting the clarity of thoughts concerning future selves.

## Introduction

A student hopes to one day write a bestselling novel, fulfilling a lifelong dream of becoming a published author. A soon-to-be father fears reliving the mistakes of his parents, determined to instead raise a child in a nurturing and supportive environment. These statements illustrate examples of future self-images or *possible selves* (Markus & Nurius, 1986). Closely related to *episodic future thinking* (Atance & O’Neill, 2001), possible selves represent thoughts that include the type of person one might become. Such thoughts disclose a person’s fears, i.e., feared possible self, and hopes i.e., hoped-for possible self, for their future identity (Markus & Nurius, 1986; Oyserman & Markus, 1990). However, possible selves are not limited to mere hopes or fears about the future; rather, they can include a sense of ‘*pre-living*’ where individuals experience what it would be like to be in the future-state (Erikson, 2007). This immersive quality can invoke emotional responses that exert an important influence on the present self (Strahan & Wilson, 2006).

The ability to clearly envision future scenarios varies across individuals (Andrade et al., 2014) which raises questions regarding the importance of future scenario immersion for psychopathological functioning. Indeed, recent research showed that the psychological “*realness*” of feared possible selves was associated to anxiety symptomology (Duffy et al., 2024). In this study, the psychological realness of feared possible selves was assessed with a measure of perceived event clarity. Clarity, also referred to as vividness, refers to how detailed and rich a mental representation is (McElwee & Haugh, 2010). Duffy and colleagues (2024) found that high anxiety was associated with clearer feared possible selves. This evidence is also consistent with insights from episodic future thinking or EFT- an overlapping psychological construct that refers to mental representations of personal future events. Deficits in EFT are suggested to be important for the development and maintenance of mental health disorders including depression, bipolar disorder, schizophrenia, OCD, eating disorders, and relevant to the present article, anxiety (Cole & Tubbs, 2022; Hallford et al., 2018). Specifically, research shows that anxiety is associated to more clear negative images and less clear positive images (Du et al., 2022; Liu et al., 2021; López-Pérez et al., 2018; Stöber, 2000).

Thus, collectively, evidence suggests that individuals with high anxiety experience greater mental immersion in negative and feared future thoughts about oneself. Previous efforts to explain this relationship have predominantly focused on attention-related biases (e.g., Du et al., 2022). For instance, according to Attentional Control Theory (Derakhshan, 2020), individuals with high anxiety are biased to selectively attend to threat-related imagery. Consequently, anxious individuals spend more time dwelling on threat-related imagery, such as their feared possible selves, which in turn enhances the clarity of these images. From this perspective, clearer feared possible selves are seen as consequences of high anxiety rather than characteristics that can contribute to anxiety symptomology (Du et al., 2022). However, the present article proposes that the clarity of feared possible selves can also contribute to anxiety through self-esteem.

Self-esteem, often described as the evaluative component of the self, pertains to individuals’ feelings of self-worth (Kernis, 2013). Individuals with high self-esteem tend to display positive self-perceptions, whereas those with low self-esteem subjectively experience more self-dissatisfaction (Kernis, 2013; Pyszczynski et al., 2004). Self-esteem is often considered a protective factor against the development of mental health disorders including depression, eating disorders and - relevant to the present article - anxiety (Mann et al., 2004). Research highlights that the relationship between anxiety and self-esteem is reciprocal whereby low self-esteem is both a predictor and a consequence of anxiety symptomology (Li et al., 2023; Sowislo & Orth, 2013). Thus, it is somewhat unsurprising that self-esteem is sometimes the focus of interventions designed to reduce anxiety (Bowles, 2017; Ntemsia et al., 2017).

The role of self-esteem has also been examined in connection with possible selves. In its simplest form, the link between self-esteem and possible selves can be understood in terms of valence, i.e., hoped-for possible selves are associated to higher feelings of self-worth and feared possible selves are associated to lower feelings of self-worth (Hulme et al., 2012; Knox et al., 1998). However, the present study proposes that the clarity of representations could exacerbate this relationship through the unique and lesser studied mechanism of ‘*pre-living’* as intimated above. Specifically, clearer mental representations of possible selves could be perceived as more psychologically *“real”* and thus will invoke emotional responses more similarly to events that occur in the present. Since positive accomplishments in the present can enhance self-esteem and negative events can reduce self-esteem, it is plausible that clearly *imagined* possible selves can have a similar impact through ‘*pre-living*’ events and their associated emotions (Baumeister et al., 2003; DeHart & Pelham, 2007). Thus, in the context of anxiety, the clarity of feared possible selves could potentially contribute towards anxiety symptomology through self-esteem since individuals with high anxiety may perceive feared possible selves as more psychologically ‘*real*’. However, to our knowledge, the relationship between self-esteem, anxiety, and the clarity of possible selves is yet to be explored.

If the clarity of feared possible selves contributes to anxiety symptomology, exploring strategies to reduce the clarity of these representations could provide insights into alleviating symptom distress. However, according to Brewin (2006), the effectiveness of interventions is not contingent on altering negative self-representations: instead, the emphasis should be on enhancing positive self-representations. Therefore, interventions focused on improving the clarity of *positive* self-images could reduce anxiety through boosting self-esteem. One way to improve the clarity of representations is through detailed elaboration (Thomas et al., 2003). An intervention that adopts this approach is the *best possible self-technique* (King, 2001). The best possible self-technique requires participants to elaborate and mentally envision their best future. Specifically, participants are asked to describe a future where everything has worked out the way they wanted it to. To date, the BPS technique has been used on various populations and has been found to increase positive affect, decrease depression, and boost optimism (Carrillo et al., 2019; Tomczyk et al., 2024). The technique can be delivered online or in-person, either individually or in small groups, and can be completed through writing, discussing one’s best future, or drawing pictures (Layous et al., 2013; Loveday et al., 2018).

The effects of the BPS technique on anxiety have not been thoroughly investigated, and to our knowledge only one study has examined this (Wu et al., 2023). Wu et al. (2023) found that the best possible self-technique did not significantly reduce anxiety in a Chinese sample. However, this result could be due to cultural differences in the tendency to elaborate on future scenarios. For instance, research shows that Chinese samples produce fewer details when imagining their future compared to samples from Western communities (Wang et al., 2011). However, to our knowledge research is yet to explore how the best possible self-technique influences anxiety with a Western sample. Considering the current need for time- and cost-effective ways of addressing the increased prevalence of anxiety disorders (Penninx et al., 2021), it is important to explore the efficacy of the BPS technique for reducing anxiety.

## The Current Study

The primary aim of the present research was to examine if self-esteem mediated the relationship between the clarity of feared possible selves and anxiety. As discussed previously, clarity could enhance the perceived realness of imagined future-self thoughts which could impact self-esteem- a construct that predicts anxiety symptomology (Li et al., 2023). It was predicted that higher anxiety would be associated to clearer feared possible selves, consistent with previous research (Duffy et al., 2024). Also consistent with previous research (e.g., Li et al., 2023), it was predicted that higher anxiety would be associated to lower self-esteem. However, due to limited research evidence, no specific hypotheses were generated regarding the relationship between self-esteem and the clarity of feared possible selves, and instead, these analyses were exploratory. A secondary aim was to explore the impact of the best possible self-technique on anxiety. Although previous research found that the BPS technique was not effective for reducing anxiety (Wu et al., 2023), to our knowledge, research is yet to examine the efficacy of the task with a western sample. For this reason, the analyses were exploratory.

## Methods

### Participants

A minimal sample size of 66 was determined based on a priori power analysis conducted on *RStudio* with the WebPower package. In total, 68 participants aged between 18–42 years were recruited (49 women, 14 men, 5 non-binary, mean age = 21.16, *SD* = 5.04) through university research participation: Participants received points as partial fulfillment to a module requirement. Participants were not asked to participate if there were receiving any current psychological or therapeutic intervention, and if English was not their first language. All participants provided informed consent and ethics approval was granted from a university Ethics Committee.

### Design

To assess the primary research question, a cross-sectional, correlational design was employed to assess the relationship between the *clarity of feared possible selves*, *self-esteem*, and *anxiety*.

To assess the secondary aim of this study – examining the effect of the best possible self-technique on anxiety - a repeated measures component was implemented whereby change in anxiety was assessed within-subjects and measured at three time-points: at baseline, after the feared possible self-task, and after the best possible self-technique. Also, to assess the interaction between task-type and baseline anxiety, participants were assigned to one of two groups (posthoc) based on *Generalised Anxiety Disorder Questionnaire* scores (Probable anxiety vs. non-probable anxiety) thus, group assignment was between-subjects. A cut off of 10 on the Generalised Anxiety Disorder Questionnaire was employed as recommended by Williams (2014). Thus, participants with a score of ≥10 were placed into a probable anxiety group and participants with a score <10 were placed into a non-probable anxiety group.

### Materials

#### Visual Analogue Scale- Anxiety

To avoid repeated use of the Generalised Anxiety Disorder Questionnaire (Spitzer et al., 2006), the present study employed the visual analogue scale-anxiety (VAS-A) to assess change in anxiety. Participants were asked to rate on a slider response bar how anxious they felt in the present moment on a scale of 0–100 (*0 = not at all anxious, 100 = very anxious*). This measure has been used in previous research and demonstrates good reliability and validity (Abend et al., 2014).

#### Generalised Anxiety Disorder Questionnaire (GAD-7)

The Generalised Anxiety Disorder Questionnaire (*GAD-7*) (Spitzer et al., 2006) is a 7-item measure assessing generalised anxiety. Items are rated on 4-point scales (*0 to 3; 0 = not at all, 3 = nearly every day*). Scores range from 0–21. Greater scores indicate greater anxiety. The GAD-7 demonstrates good reliability and validity (Byrd-Bredbenner et al., 2020).

#### The Robson Self-concept Questionnaire (SCQ)

The Robson Self-concept Questionnaire (SCQ; Robson, 1989) is a 30-item measure assessing self-esteem (e.g., “*I can like myself even if others don’t*”). Participants were asked to rate their self-esteem over the last two weeks. Items are rated on 8-point scales (0 to 7; 0 = completely disagree, 7 = completely agree). Greater scores indicate higher self-esteem. The questionnaire demonstrates good reliability and validity (Ghaderi, 2005).

#### Feared Possible Self Clarity

First, the participants were introduced to the concept of feared possible selves and were presented with examples (*homeless, alone, and arrogant)*. Participants then described what they feared to be in the future and were asked a question assessing clarity (“Images of this thought are hazy, not clear at all*“) rated on a 5-point Likert scale (1 to 5; 1 = completely disagree (*images are very clear*), 5 = completely agree (*images are not clear*). The participants could record as many feared possible selves as they could think of, but they had to record a minimum of one to move on. Clarity was assessed for each feared possible self and average clarity ratings were calculated for each participant.

#### Best Possible Self Technique

The best possible self-technique (King, 2001) is a writing exercise that involves writing about one’s best possible future self. Specifically, participants were instructed “*Next, we want you to imagine yourself in a future where everything has gone right. You’ve accomplished your goals, and as a result, you’ve become your best possible self. What would your life look like? How would you spend your time? Who would be by your side? Please write as much detail as you can think of below. Do not worry about grammar or spelling*.” Participants then responded by typing a detailed response in a survey text box.

### Procedure

The study was presented online and the participants could complete it in a place of their choosing. Following the consent procedure, the participants completed the GAD-7 and the SCQ. The order in which these questionnaires were presented was randomised. The participants then completed the feared possible selves task followed by the best possible self-technique. Current anxiety was assessed at three time points 1] at the start of the study (baseline) 2] after the feared possible self-task (T1) 3] after the best possible self-task (T2).

### Data Analysis

Data was analysed on RStudio version 4.2.1 and Jamovi version 2.6.13. Correlation analyses were used to assess the relationship between anxiety, clarity, and self-esteem. Mediation analyses were conducted using the *mediation* package in *r* with 5000 bootstrapped confidence intervals. To assess change in anxiety, a mixed methods analysis was employed in Jamovi. Differences in the ratings of VAS-A were analysed using a linear mixed-effects model with restricted maximum likelihood estimation (REML). The model included random intercepts. Group assignment (Probable anxiety, non-probable anxiety) and time were included as fixed effects, along with their interaction. The models were specified as follows: anxiety ∼ 1 + group + time + (1 | participantID). https://osf.io/rtaep/?view_only=ee6f739a05db4428aba078f52210f4b4

## Results

### Correlational Analyses and Mediation

On average participants recorded 2.22 feared possible selves (*SD* = 1.20). Example descriptions of feared possible selves are presented in Appendix A. Reliability statistics were excellent for both the *Generalised Anxiety Disorder Questionnaire* (*a* = .87) and *the Self-Concept Questionnaire* (*a* = .88). Spearman’s correlations were employed to assess the relationship between anxiety, self-concept, and the clarity of feared possible selves. First, there was a significant relationship between anxiety and the clarity of feared possible selves (*r*(66) = .33, *p* = .006) showing that anxiety increased as clarity increased. Next, there was a significant relationship between anxiety and self-esteem (*r*(66) = -.53, *p* < .001) whereby self-esteem decreased as anxiety increased. Finally, there was a significant relationship between self-esteem and the clarity of feared possible selves (*r*(66) = −.39, *p* = .001) whereby the clarity of feared possible selves decreased as self-esteem increased.

A mediation analysis was performed to investigate whether the relationship between clarity and anxiety was mediated by self-esteem. The outcome variable in the analysis was anxiety, the predictor variable was clarity, and the mediator was self-esteem. First, clarity significantly predicted anxiety (the total effect) *b* = 1.57, 95% CI [.45, 2.70], *p* = .008. Once self-esteem was entered into the model, clarity did not significantly predict anxiety (the direct effect) *b* = .77, 95% CI [−.48, 2.01], *p* = .235. However, the indirect effect via self-esteem was significant *b* = .80, 95% CI [.13, 1.84], *p* = .008. Thus, self-esteem fully mediated the relationship between clarity and anxiety.

### Change in Anxiety

Estimated marginal means with 95% CI are presented in Table 1. Examples of best possible self-descriptions are presented in Appendix A. The results showed a significant main effect of Time (Baseline, T1, T2) *F*(2) = 25.22, *p* < .001 and Group (Probable Anxiety, Non-Probable Anxiety) *F*(1) = 26.73, *p* < .001. However, these effects were superseded by a significant *Time X Group* Interaction *F*(2) = 5.05, *p* = .008, see Figure 1. Bonferroni corrected post hoc comparisons showed that at baseline the participants in the probable anxiety group reported significantly higher anxiety compared to participants with non-probable anxiety *p* = .004. After the feared possible self-task, the participants in the probable anxiety group reported an increase in anxiety -although this was not significant after Bonferroni corrected post hoc analyses *p* = .067. The participants in the non-probable anxiety group reported no change in anxiety following the feared possible self-task *p* = 1. After the BPS technique, the participants in the probable anxiety group reported a significant decrease in anxiety from baseline *p* = .007. Likewise, the participants in the non-probable anxiety group also reported a significant decrease in anxiety from baseline to time 2 following the BPS technique *p* < .001

**Table 1.** Estimated Marginal Means With 95% Confidence Intervals.

	Probable Anxiety Mean (CI)	Non-probable Anxiety Mean (CI)
Baseline	52.6 (45.4, 59.7)	32.9 (25.4,40.5)
Post-Feared task	60.2 (53.1, 67.3)	28.8 (21.3, 36.4)
Post-BPS task	43.1 (36,50.2)	20.7 (13.1, 28.2)

Note: Feared task = Feared Possible Self Task, BPS task = The Best Possible Self Task.

**Figure 1.**
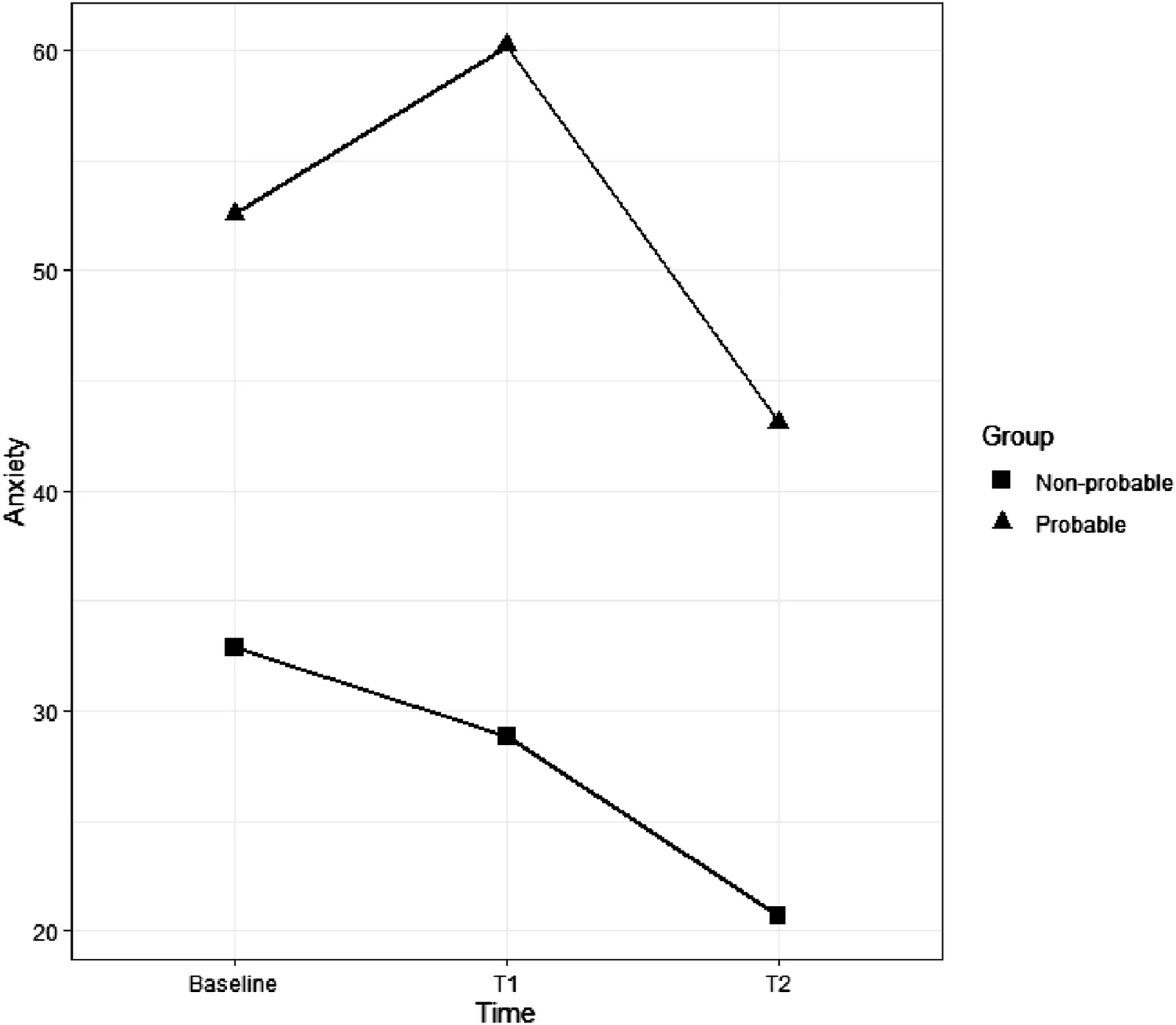
Change in VAS-A Scores as a Function of Time and Group. Notes: Probable= Probable anxiety, non-probable= non-probabale anxiety.

## Discussion

The main aim of the present research was to investigate whether self-esteem mediates the relationship between the clarity of feared possible selves and anxiety. The secondary exploratory aim was to assess the preliminary impact of the best possible self-task on anxiety- assessed at three time points: baseline, post feared possible-self (T1), post best possible self (T2).

First, following our predictions, it was found that self-esteem mediated the relationship between the clarity of feared possible selves and anxiety. This finding highlights a potential mechanism through which the clarity of feared possible selves influences anxiety. Previous attempts to explain the relationship have relied on attention-related explanations (e.g., Du et al., 2022): For instance, according to Attentional Control Theory, anxious individuals are biased to selectively attend to negative representations which enhances their perceived clarity (Derakhshan, 2020). From this perspective, mental image clarity is seen as a consequence of high anxiety. However, results from the present study demonstrate that the clarity of feared possible selves could also contribute to anxiety symptomology through self-esteem. As discussed in the introduction, clarity represents the level of immersion individuals experience in a future-self thought. As such, clearer possible selves could be perceived as more psychologically “*real*” and thus will invoke emotional responses more similarly to events that occur in present reality. Since negative events can reduce self-esteem (DeHart & Pelham, 2007), it was proposed that clearly imagined feared possible selves could have a similar impact through pre-living. The results of the present study provide preliminary evidence in support of this proposed mechanism and demonstrate how self-related constructs can interact with other variables to contribute to anxiety symptomology. Nonetheless, we note that the Attentional Control Theory account of anxiety is not disputed by this mechanism or our findings, and we anticipate that both approaches will reap improvements for theoretical understanding of anxiety and its treatments.

Next, to examine whether and how the best possible self-technique effects in-the-moment anxiety, a repeated measures design was adopted whereby post-task anxiety could be compared with prior states of anxiety (e.g., baseline). In line with predictions, it was found that the best possible self-task significantly reduced anxiety for both groups. As discussed previously, we hypothesise that the best possible self-task can reduce anxiety through increasing the clarity of hoped-for possible selves which could have boosted self-esteem. However, since change in clarity and self-esteem was not assessed in our second research question, future research should examine this effect further to determine the mechanism responsible for these effects. These findings have potential implications for therapeutic interventions aimed at reducing anxiety. However, before the BPS technique can be delivered to a clinical group, more research is needed to understand its long-term efficacy, as well as potential moderators to its efficiency. Interestingly, the feared possible self-task was found to increase anxiety for participants in the probable anxiety group (although not significantly). This is likely because participants with high anxiety imagine feared future images with increased clarity so the representations will feel more real (Mathews et al., 2013).

## Limitations and Future Directions

Although the present article provides valuable insights into anxiety and self-related cognition, it is important to acknowledge some important limitations. First, the present article relied on student participation thus, it is important to consider the representativeness of the sample when interpreting the results. In addition, demographic information including ethnicity was omitted from data collection. This was intentional since demographic differences was not the scope of the present article, however, future research should explore whether these findings are relevant to all demographics. Also, since a non-clinical sample was recruited, the present article cannot generalise these findings to clinical populations. Instead, the results of the present study can only generalise to samples with *probable anxiety,* (who may or may not have a concomitant diagnosis of an anxiety disorder). Future research should replicate the present study with different populations including a clinical sample of anxious participants.

Additionally, regarding the best possible self-task, ratings of clarity or post self-esteem were not measured; thus, we do not know if the best possible self-task increased the clarity of hoped-for identities and boosted self-esteem or if it had a different effect on participants perceptions of their future selves. Future research should investigate if the best possible self-task does increase the clarity of hoped-for identities and boosts self-esteem which, if true, may explain why it was effective at reducing anxiety in the present study. These questions are currently being investigated by the study authors. In addition, since the present study presented cross-sectional data, the repeated-measure effects of the best possible self-task could not be established and can only be seen as preliminary, yet intriguing. Specifically, further research is needed to establish whether the effects of the task would be present after a longer period of time or if repeated use (a larger “dosage”) is required. Future applied research in this area should explore the possible benefits longitudinally. These are important areas for future research to explore since the BPS technique could have important clinical implications. Specifically, since it is a task that can be delivered online without the need for clinician, it is relatively cost-effective and potentially more accessible than usual interventions (Bekker et al., 2017).

## Conclusion

In summary, the present article aimed to explain a recently found relationship between anxiety and the clarity of feared possible selves (Duffy et al., 2024). The results herein demonstrated a significant association between the clarity of feared possible selves and anxiety - a relationship found to be mediated, or explained, by self-esteem. Thus, clearer representations of feared possible selves could contribute to anxiety symptomology because they engender low self-esteem. In addition, the best possible self-task was found to significantly reduce anxiety. Further research is now needed to determine the implications of the best possible self-task in clinical groups and if its effects can be maintained after one session or if repeated “doses” are required. Furthermore, future research should explore how the best possible self-task works to reduce anxiety for instance: Does it increase the clarity of hoped-for identities which then boosts self-esteem? Overall, the present article highlights the significance of the future-self perceptions for the maintenance and development of anxiety and points toward a much-needed low-cost intervention for a common mental health disorder.

## Data Availability

Data is available on the Open Science Framework Repository, and can be accessed via the following link: https://osf.io/rtaep/?view_only=ee6f739a05db4428aba078f52210f4b4

## Appendix A

### Examples of Feared Possible Selves

**Example 1:** “Avoid being bad at social interactions”

**Example 2:** “Someone who hasn’t reached their full potential”

**Example 3:** “Being left with no family”

### Examples of Best Possible Selves

**Example 1:** “I graduated university with a good grade and I’m on the path to having a job in mental health/counselling. I am still with my boyfriend and I am still close with my best friends I have now. I could still live in [home town] but I’m also happy to move somewhere else. I spend my time going to gigs and travelling with my boyfriend/my friends as well as visiting home.”

**Example 2:** “I would have a slow-paced life, where i can take my time to rise in the morning, feel well rested and not feel rushed throughout the day. I would work at my yoga studio and hold spaces for people such as in women circles and meditation classes. I would still be writing poetry and making art, but for the purpose of my soul expression rather than for others to like it. I would love myself so unconditionally that i don’t rely on the love of others in order to feel good enough about myself. I have the time to nourish my body with whole foods, and continue my daily meditation and yoga classes. Spending time in nature and in solitude is a big priority for me and i make time for this regularly. I don’t need to earn a lot of money, just enough to live comfortably and have a good work/ life balance. I will have a little home of my own that feels like a sanctuary. i feel safe and comfortable there. I am surrounded by people who love and care for me. People who support my growth and healing and I’m able to lean into support from these people whilst also putting boundaries in place with those who don’t support my growth and healing. I have the time and space in my life to be able to travel but also always have a home to come back to and feel grounded again.”
